# *Quercus ilex* Phyllosphere Microbiome Environmental-Driven Structure and Composition Shifts in a Mediterranean Contex

**DOI:** 10.3390/plants11243528

**Published:** 2022-12-15

**Authors:** Alessia Postiglione, Antonello Prigioniero, Daniela Zuzolo, Maria Tartaglia, Pierpaolo Scarano, Maria Maisto, Maria Antonietta Ranauda, Rosaria Sciarrillo, Sofie Thijs, Jaco Vangronsveld, Carmine Guarino

**Affiliations:** 1 Department of Science and Technology, University of Sannio, via de Sanctis snc, 82100 Benevento, Italy; 2 Environmental Biology, Centre for Environmental Sciences, Hasselt University, Agoralaan, Building D, 3590 Diepenbeek, Belgium; 3 Department of Plant Physiology and Biophysics, Institute of Biological Sciences, Maria Curie-Skłodowska University, Akademicka 19, 20-033 Lublin, Poland

**Keywords:** next-generation biomonitoring, *Quercus ilex*, core microbiome, functional traits, fungi, bacteria, particulate matter, phyllosphere

## Abstract

The intra- and interdomain phyllosphere microbiome features of *Quercus ilex* L. in a Mediterranean context is reported. We hypothesized that the main driver of the phyllosphere microbiome might be the season and that atmospheric pollutants might have a co-effect. Hence, we investigated the composition of epiphytic bacteria and fungi of leaves sampled in urban and natural areas (in Southern Italy) in summer and winter, using microscopy and metagenomic analysis. To assess possible co-effects on the composition of the phyllosphere microbiome, concentrations of particulate matter and polycyclic aromatic hydrocarbons (PAHs) were determined from sampled leaves. We found that environmental factors had a significative influence on the phyllosphere biodiversity, altering the taxa relative abundances. Ascomycota and Firmicutes were higher in summer and in urban areas, whereas a significant increase in Proteobacteria was observed in the winter season, with higher abundance in natural areas. Network analysis suggested that OTUs belonging to Acidobacteria, Cytophagia, unkn. Firmicutes(p), Actinobacteria are keystone of the *Q. ilex* phyllosphere microbiome. In addition, 83 genes coding for 5 enzymes involved in PAH degradation pathways were identified. Given that the phyllosphere microbiome can be considered an extension of the ecosystem services offered by trees, our results can be exploited in the framework of Next-Generation Biomonitoring.

## 1. Introduction

The phyllosphere provides a peculiar surface involved in various plant-environment interaction processes [1,2] and, on a global scale, its surface area is comparable to the entire land surface of the planet [3]. Hence, in recent years, the phyllosphere has become a central topic in various scientific fields, especially urban forestry, plant physiology, and environmental microbiology [4]. This surface, often featured by morphological traits depictable by cuticle, stomata, and trichomes, offers available spaces involved in different aspects of ecosystem functioning, acting as a contact interface between plants, the external environment, and organisms belonging to all trophic levels [4,5]. The surface of leaves, although representing a hostile environment for some species, hosts a large and diverse microbial community [6,7,8]. These organisms, of which bacterial and fungal symbionts are the most abundant, find a habitat of spaces, protection, and exudates, due to which they can thrive among the leaf topological features and structures, such as the stomata, veins, and trichomes [9,10]. The epiphytic microbiomes, which aggregate and colonize the surfaces of leaves, interact with the host, mediating plant responses to stressful factors, biosynthesizing plant hormones, or contributing to the degradation of on-leaf pollutants [11,12], to the extent that the plant-phyllosphere-microbiome system can be considered a holobiont [13]. To date, epiphytic phyllosphere communities associated with several plant species have been identified, both for herbaceous and tree hosts [14,15,16,17,18,19,20]. It is now well known how the plant genotype, its immune system, and its phenotypic features determine the shaping of phyllosphere epiphytic communities found on leaves, mediated by the presence and production of exudates and specific aspects of leaf topology [21]. In turn, the microbiome affects plant physiology by mediating plant responses to biotic and abiotic stresses or degrading organic pollutants [18,19,21,22]. Changes in the biodiversity and abundance of the phyllosphere microbiome communities have been observed when external environmental pressures occur [18,19,20,23,24,25]. The composition of the phyllosphere microbiome was described to be subject to seasonal changes of temperature, humidity, and irradiance, and environmental conditions linked to biotic and abiotic stress factors [23,25,26,27,28]. Many authors reported that the presence of airborne pollutants is one of the factors driving changes in the phyllosphere microbiome composition [24,29]. The contexts where air pollution pressure is the highest are urban environments, and the presence of plants in these highly anthropized environments also suggests a strong selective factor on the phyllosphere microbiome of host plant species [16,18].

In the last decades, with the developments of urban forestry and ecosystem services concepts, the benefits attributed to urban green and urban forests were intensively investigated [30,31]. Among the ecosystem services described, sequestration and retention of atmospheric pollutants by the plant canopy leaves is a relevant topic [32]. It has already been proven that trees and shrubs can mitigate the pollution produced by anthropic activities in the atmosphere of urban areas, efficiently removing particulate matter, both coarse (PM_10_) and fine (PM_2.5_) [33,34,35,36], and the polycyclic aromatic hydrocarbons eventually bound to it [37]. Perennial trees and shrubs, especially evergreen and sclerophyllous ones, with leaves remaining on the canopy for longer than a single growing season, proved tolerant of abiotic stress and increased ability to retain atmospheric pollutants [38,39]. Moreover, the presence of functional traits, such as dense trichomes and thick cuticular layers, proved high performance of pollutants sequestration and retention [37,40,41]. However, only recently has the degradation ability of phyllosphere microorganisms been investigated, and it has been recognized as an extension of the ecosystem services offered by trees because it can contribute to a reduction of air pollution [12,42,43]. Plant-associated microbiomes are structured by environmental conditions. However, it is not yet clear to which extent environmental factors can alter the phyllosphere microbiome, with consequences for both the ecosystem services it provides and its relationship to the host genotype. Although numerous studies started exploring microbial co-occurrence in a variety of plant species and environments [44], our understanding of leaf microbial diversity and its potential ecological relevance is still limited [45]. Moreover, the concept of Next-Generation Biomonitoring (NGB) proposes the use of networks of species interactions for ecosystem monitoring [46]. Given the above, we attempted to add new knowledge potentially useful in NGB for *Quercus ilex* L., a Mediterranean tree species (widely adopted in urban forests) well studied in atmospheric PAHs and trace elements biomonitoring [35]. The phyllosphere microbiome of this species and how it is influenced by environmental factors has been less investigated to date, despite their ecological and applicative relevance.

Hence, with this study, we first attempted to define the inter- and intra- domain features of the *Q. ilex* phyllosphere microbiome.

We also wondered whether it might exhibit specific host association patterns in a defined environment under seasonal and airborne PM_10_ levels and PAHs pollution pressures. Furthermore, we also questioned what role microbial communities of *Q. ilex* phyllosphere might play in the degradation of PAHs. In order to address these questions, our study aimed to explore (i) the colonization of the leaf surfaces by bacteria and fungi, (ii) in-depth metagenomic study of the epiphytic microbiome (both fungi and bacteria), and (iii) the research of genes coding for enzymes involved in the degradation pathway of polycyclic aromatic hydrocarbons.

## 2. Results

### 2.1. Visualization of Epiphytic Fungal and Bacterial Microbiome

**Figure 1:**
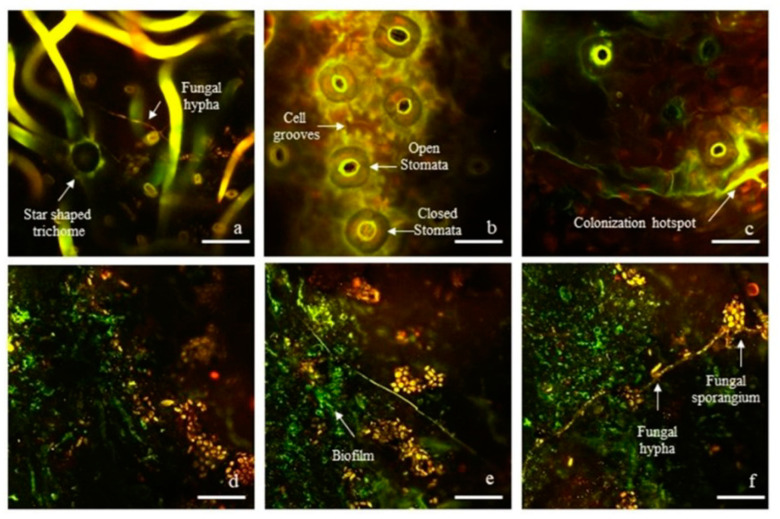
Panel showing phyllosphere microorganisms arrangements with regard to *Q. ilex* leaf functional traits (cuticle, stomata, and trichomes). Images have been obtained through the use of LIVE/DEAD™ BacLight™ Bacterial Viability Kit and two-photon excitation microscope (Ultima investigator 2-photon microscope—Bruker, MS, USA). Images (**a**–**c**) referred to abaxial leaves surfaces; images (**d**–**f**) referred to adaxial leaves surfaces. The images were obtained by merging the two emission filters, in green and red, to allow the visualization of live and dead (bacterial and fungal) cells, respectively, highlighted by the dyes used (Syto9 and propidium iodide). Scale bar = 20 µm (**b**–**f**); Scale bar = 50 µm (**a**).

The abaxial side of leaves of *Q. ilex* showed recurrent and specific localization of microorganisms at the stomatal openings, indicating a strong signal of living cells (Figure 1a–c). Microscopic analyses also highlighted clear signaling on the branches of the abaxial stellate trichomes and at their base (Figure 1a). Colonization hotspots were obvious in the lower grooves of epidermal cells (Figure 1b,c). In all the cases just described, there was a dark red background, corresponding to the leaf chlorophyll autofluorescence. The chlorophyll signal, from the chloroplasts of epidermal cells, cannot be removed as it has an autofluorescence emission peak around 685 nm, close to the maximum emission peak of the Propidium Iodide (PI) at 635, both of which were detectable in the red channel. The presence of bacterial biofilms on the adaxial leaf surfaces was evident, and it was of an exopolysaccharide nature, in which micro-organisms remain embedded (Figure 1d–f). Samples also indicated high fungal proliferation on the adaxial sides highlighted in orange-red (Figure 1d–f). Although fungal structures were present mainly on the upper sides of leaves rather than the abaxial ones, hyphae and spores were also found among the stellate trichomes intricate network, which is typical for *Q. ilex* leaves (Figure 1a).

### 2.2. Particulate Matter and PAHs Concentrations

The concentrations of coarse particulate matter extracted using distilled water in *Q. ilex* leaves ranged from 91,628.54 ± 959.47 ng cm^−2^ to 37,789.85 ± 4549.16 ng cm^−2^ (Table 1). In both seasons, the highest concentrations were found in samples from the urban environment, while the lowest concentration was found in leaves sampled in the natural environment during the winter season (Table 1). On the filters obtained from chloroform extractions, PM_10_ concentrations were found to be lower than those extracted with distilled water, measuring in the range of 38,926.46 ± 755.81 ng cm^−2^ to 21,022.88 ± 1443.39 ng cm^−2^ (Table 1). The ANOVA analysis showed that the concentrations found on leaves collected in the winter in an urban environment were similar to all the others, while the remaining samples were ordered as: NA Summer > UA Summer > NA Winter (Table 1). Benzo(a)pyrene was found in all sampled leaves and, as with particulate matter, the concentrations of the pollutant found from extractions in distilled water were higher than those obtained using chloroform (Table 1). Benzo(a)pyrene concentrations from extractions in distilled water showed no significant differences between samples, whereas for those extracted in chloroform, the samples were ordered as: NA Summer > UA Winter = UA Summer, and NA Winter was not significantly different from all the other samples (Table 1). Only in samples taken from natural areas were appreciable concentrations of Benzo(a)anthracene extracted with chloroform, measuring higher in summer than in winter (Table 1). In contrast, Benzo(b)fluoranthene was detected only in the aqueous extracts of samples taken in the winter from urban areas, as well as in the water and chloroform extracts of leaves sampled in the summer from natural areas (Table 1). Benzo(j)fluoranthene was extracted in concentrations above the instrumental detection limit only in UA Winter samples after extraction with distilled water, while detectible concentrations of Indeno[1,2,3-cd]pyrene were found in NA Summer samples after extraction with chloroform (Table 1). Finally, Benzo[k]fluoranthene and Dibenz[a,h]anthracene were not detected in any sample using either solvent (Table 1).

### 2.3. Relative Abundance of Phyla and Classes of Epiphytic Microorganisms

The surface area of *Q. ilex* leaves sampled in the winter in urban areas ranged from 235.53 cm^2^ to 292.34 cm^2^, while those sampled in natural areas during the winter season were in the range of 304.88 cm^2^ to 374.30 cm^2^. The surfaces of leaves sampled in the summer in urban and natural areas varied from 303.18 cm^2^ to 275.61 cm^2^ and from 310.18 cm^2^ to 370.03 cm^2^, respectively. The average epiphytic DNA extracted from plant material taken during the winter sampling campaign was 12.51 ± 2.96 ng cm^−2^ in urban areas and 10.00 ± 2.00 ng cm^−2^ in natural areas. The average amounts of DNA extracted by washing the leaf surfaces collected in the summer in urban and natural areas were 7.27 ± 1.17 ng cm^−2^ and 11.00 ± 3.00 ng cm^−2^, respectively. After taxonomic profiling of the samples, reads attributed to OTUs (Operational Taxonomic Units) that did not belong to the Bacteria and Fungi kingdoms were removed, resulting in a deletion ranging from 3.62% to 11.65% for Phylum and between 3.48% and 11.63% for Classes.

**Figure 2:**
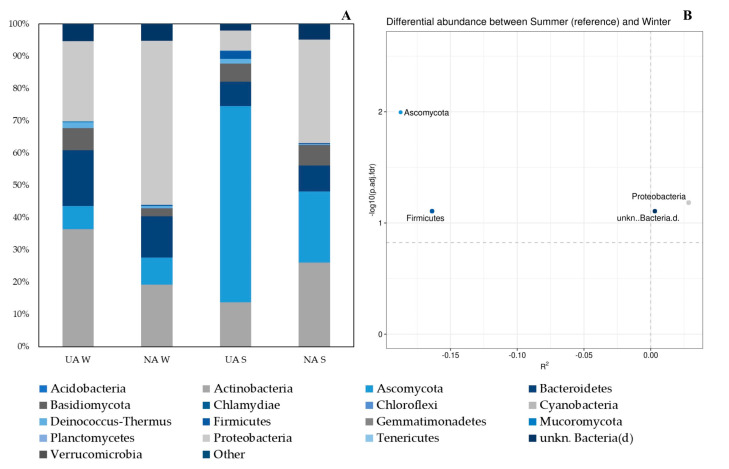
(**A**) Relative average abundance of phyllospheric bacterial and fungal taxa at Phylum level. (**B**) Differential Abundance Analysis (DAA) plot (based on ZicoSeq (*GUniFrac* package). The plot shows the *y*-axis as the log10 (adjusted) *p*-value and the *x*-axis as the signed R^2^, which indicate the association direction with the covariate (− and + with urban and natural areas, respectively). The names of differential taxa passing a specific significance cutoff (permutation-based FDR-adjusted *p*-Values = 0.1) are printed on the figure. (**A**) Only taxa with an average abundance ≥ 0.01% in at least one of the four groups (Urban Area W, Natural Area W, Urban Area S, Natural Area S) of samples are shown. Samples are grouped according to sampling season (W = winter or S = summer) and environmental features (Urban Area or Natural Area).

The phylum showing the highest average relative abundance on the surface of samples taken in urban areas in the winter was Actinobacteria (36.34%), followed by Proteobacteria (24.86%) and Bacteroidetes (17.38%) (Figure 2).

In the same season, on the leaves from natural areas, the most represented phyla were the same as those found in urban areas, but in a different order: Proteobacteria (50.82%) > Actinobacteria (19.19%) > Bacteroidetes (12.89%) (Figure 2). In both cases, the most represented fungal phylum on average was Ascomycota, although with much lower percentages than the bacterial phyla (Figure 2). In the summer, the taxonomic composition was different: in urban areas, 60.76% of the organisms found on leaf surfaces belonged to the phylum Ascomycota (fungal), followed by Actinobacteria and Bacteroidetes, which accounted for an average of 13.76% and 7.56%, respectively (Figure 2). The average relative abundance of Proteobacteria (31.98%) was highest on leaves sampled in the summer in natural areas, followed in decreasing order by Actinobacteria (25.95%) and Ascomycota (22.06%). At the phylum level, DAA (Figure 2B) showed that four taxa (Firmicutes, Proteobacteria, unkn.Bacteria.d., and Ascomycota) significantly changed between the summer and winter samples (ZicoSeq; p.adj.fdr <= 0.1). Since R^2^ indicates whether the abundance decreases (−) or increases (+) with the covariate (in our case, corresponding to the area type: urban/natural); we also observed that a cofounder effect on taxa abundance occurred. Among the differentially abundant taxa, Ascomycota and Firmicutes abundance is higher in urban areas, whereas Proteobacteria and unkn.Bacteria.d. increase in natural areas.

**Figure 3:**
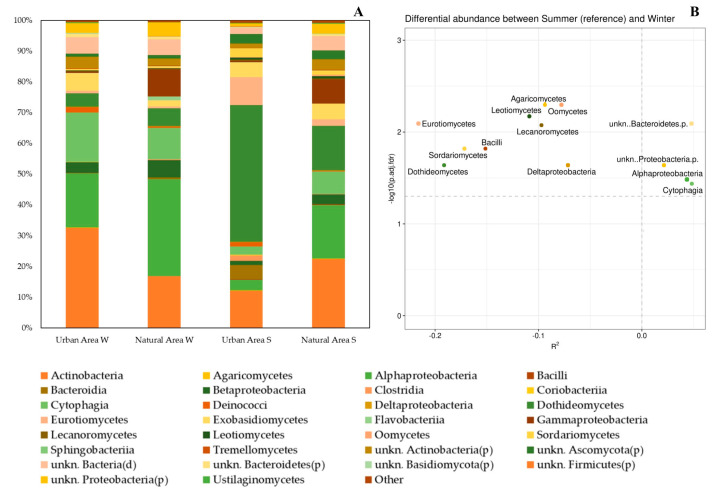
(**A**) Relative average abundance of phyllospheric bacterial and fungal taxa at Class level. (**B**) Differential Abundance Analysis (DAA) plot (based on ZicoSeq (*GUniFrac* package). The plot shows the *y*-axis as the log10 (adjusted) *p*-value and the *x*-axis as the signed R^2^, which indicate the association direction with the covariate (− and + with urban and natural areas, respectively). The names of differential taxa passing a specific significance cutoff (permutation-based FDR-adjusted *p*-Values = 0.1) are printed on the figure. (**A**) Only taxa with an average abundance ≥ 0.10% in at least one of the four groups (Urban Area W, Natural Area W, Urban Area S, Natural Area S) of samples are shown. Samples are grouped according to sampling season (W = winter or S = summer) and environmental features (Urban Area or Natural Area).

The three classes with the highest average relative abundances in leaf samples taken in the winter, both in urban and natural areas, were Actinobacteria, Alphaproteobacteria, and Cytophagia (Figure 3). What differentiated the two environmental contexts were the percentages in which these three classes were distributed: in urban areas, they were in descending order as Actinobacteria (32.59%), Alphaproteobacteria (17.70%), and Cytophagia (16.04%), while in natural areas the descending order was Alphaproteobacteria (31.58%), Actinobacteria (16.88%), and Cytophagia (10.14%) (Figure 3). From the samples taken in urban areas during the summer, slightly less than half of the organisms belonged to the class Dothideomycetes (44.52%), while the other two most represented classes were Actinobacteria and Eurotiomycetes, to which 12.18% and 9.00% of the taxa found belonged, respectively (Figure 3). On the leaf surfaces sampled in the summer from natural areas, the class with the highest average relative abundance was Actinobacteria (22.41%), followed by Alphaproteobacteria (17.46%) and Dothideomycetes (14.51%) (Figure 3).

DAA (Figure 3B) showed that 13 taxa (Agaricomycetes, Dothideomycetes, Eurotiomycetes, Lecanoromycetes, Leotiomycetes, Oomycetes, Sordariomycetes, Alphaproteobacteria, Bacilli, Cytophagia, Deltaproteobacteria, unkn.Bacteroidetes.p., and unkn.Proteobacteria.p.) are differentially abundant in relation to the season. Moreover, additive effects of area type on species abundance occur. Our data report that Alphaproteobacteria, Cytophagia, unkn.Bacteroidetes.p., and unkn.Proteobacteria.p. abundance increase in natural areas, while fungal taxa characterize urban areas.

### 2.4. Epiphytic Microorganism Co-Occurrence Network

**Figure 4:**
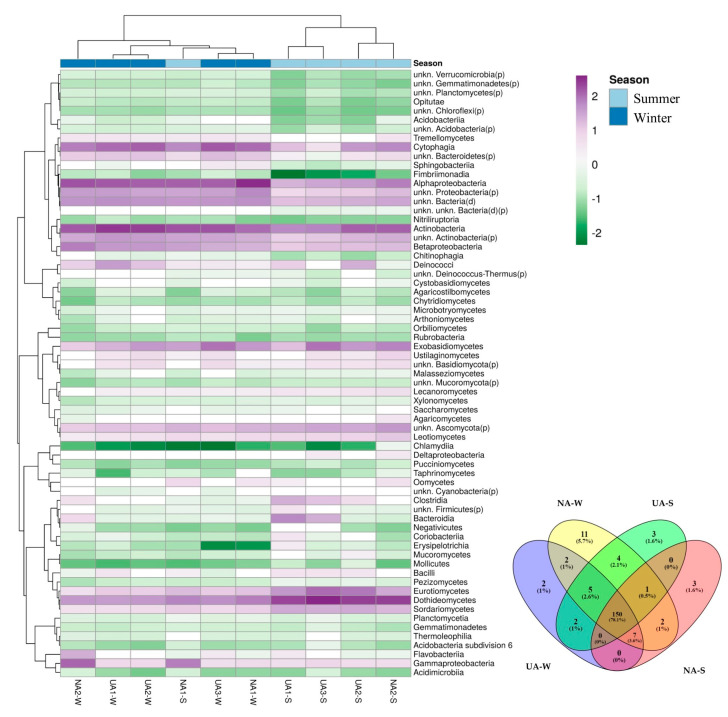
(**A**) Heatmap with clustering of OTUs in *Q. ilex* phyllosphere. The columns correspond to the samples, while the rows correspond to the OTUs. Column are centered; unit variance scaling is applied to columns. Both rows and columns are clustered using correlation distance and complete linkage (67 rows, 10 columns). Columns (samples) are clustered according to season (Summer and Winter). (**B**) Venn diagram showing distribution of microbial OTUs between samples collected in different seasons (winter = W; summer = S) and environments (urban areas = UA; natural areas =NA). Heatmap was created using ClustVis, a tool for visualizing clustering of multivariate data [48]; Venn diagram was built using “Venny” online tool [49].

Considering the phyllosphere relationships of the different samples (Figure 4A), a clear distinction based on the season (summer and winter) was observed, while the samples could not be distinguished based on the sampling area (natural and urban). The taxa Eurotiomycetes, Dothideomycetes, and Sordariomycetes appeared to be characteristic for the summer samples. Microbial taxa mainly characterizing the summer samples were Cytophagia and Alphaproteobacteria, while Actinobacteria were consistent over the season progression.

More than 78.1% of the microbial taxa (150 OTUs) are shared between samples (Figure 4B), and they represent the core microbiome of *Q. ilex* in the investigated environmental context. Furthermore, the total number of OTUs unique for the winter phyllosphere was 15 OTUs, of which 2 were shared between the urban and natural environments, while 6 OTUs were unique for the summer phyllosphere, of which none were shared between the 2 environments (Figure 4B).

**Figure 5:**
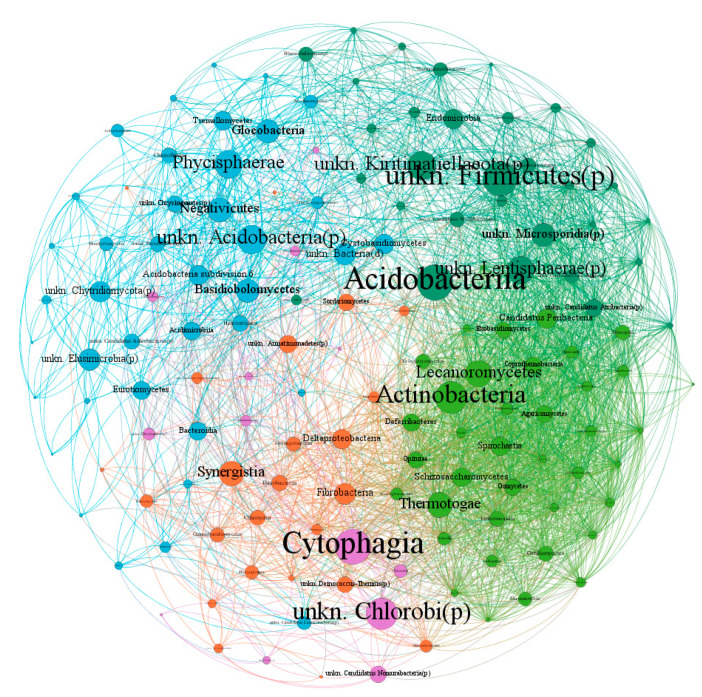
Network analysis revealing the co-occurrence patterns between OTUs (class level). The nodes were colored according to modularity class. The connection between nodes represents a strong (Spearman’s correlation coefficient ρ > 0.5) and significant (*p*-Value < 0.05) correlation. The size of each node is proportional to the proximity of a node to all other nodes in the network (that is, Betweenness Centrality).

The microbial network (Figure 5) consisted of 146 nodes (OTUs) linked by 3164 edges, where positive correlations were more numerous compared to negative ones (Table S1 in Supporting Information). The average network distance between all pairs of nodes (average path length) was 2.34 edges, with a clustering coefficient (that is, how nodes are embedded in their neighborhood and, thus, the degree to which they tend to cluster together) of 0.614 and a modularity index of 0.44 (values >0.4 suggest that the network has a modular structure [47]). Overall, the microbial network comprised highly connected OTUs (~22 edges per node, Table S1 in Supporting Information). The whole network was grouped into five major modules. Module I and Module II each included 26.7% of the nodes in the entire network (Figure S1 in Supporting Information), which corresponds to 39 OTUs. Module III and IV occupy 21.2% and 16.4% of the nodes, respectively, whereas module V consisted of 8.9% of OTUs.

Some keystone OTUs were identified from the co-occurrence network of the *Q. ilex* phyllosphere, based on BC values. Acidobacteriia, Cytophagia, unkn. Firmicutes(p), Actinobacteria, unkn. Chlorobi(p), unkn. Kiritimatiellaeota(p), Phycisphaerae, unkn. Lentisphaerae(p), Lecanoromycetes, Thermotogae, Synergistia, Negativicutes, and Basidiobolomycetes were identified as keystone class candidates (Figure 5).

### 2.5. Functional Metagenomics

Functional metagenomic analysis carried out on the epiphytic microorganisms of the *Q. ilex* leaves sampled in the two seasons (winter and summer) and in the two different environmental contexts (urban and natural) made it possible to identify 83 different genes whose coding is related to 5 enzymes involved in the pathways of PAH degradation (pathway code: ec00624; [50]): protocatechuate 3,4-dioxygenase (EC 1.13.11.3), protocatechuate 4,5-dioxygenase (EC 1.13.11.8), phthalate 4,5-dioxygenase (EC 1.14.12.7), and 4,5-dihydroxyphthalate decarboxylase (EC 4.1.1.55) (Table S2 in Supporting Information).

**Figure 6:**
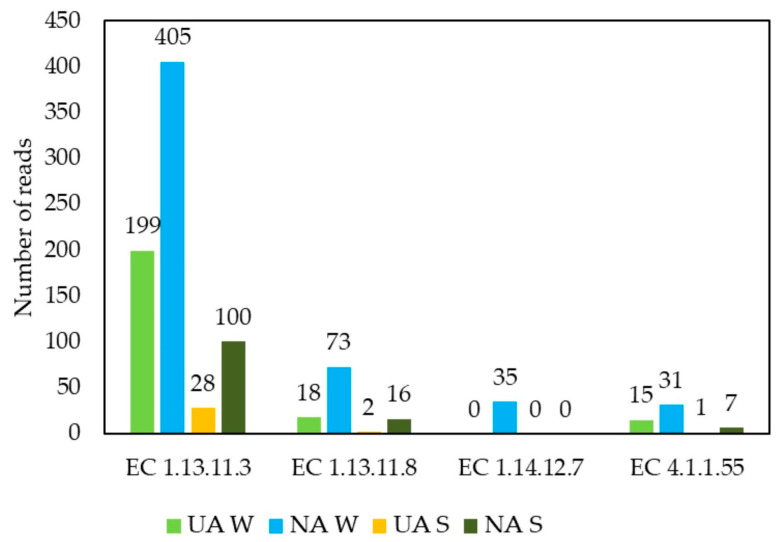
Barplot showing the average value, for each sample type (Urban Areas Winter-UA W; Natural Areas Winter-NA W; Urban Areas Summer-UA S; Natural Areas Summer-NA S), of reads number for genes involved in the synthesis of the enzymes protocatechuate 3,4-dioxygenase (EC 1.13.11.3), protocatechuate 4,5-dioxygenase (EC 1.13.11.8), phthalate 4,5-dioxygenase (EC 1.14.12.7), 4,5-dihydroxyphthalate decarboxylase (EC 4.1.1.55) revealed by the functional metagenomic analysis carried out on the microorganisms extracted from the leaf samples.

Using a barplot, it was possible to see that the average reads for each enzyme were higher in the winter samples from the natural areas (NA W) (Figure 6). Considering the average number of reads related to protocatechuate 3,4-dioxygenase, protocatechuate 4,5-dioxygenase, and 4,5-dihydroxyphthalate decarboxylase enzymes, the other samples are ordered as: UA W > NA S > UA S (Figure 6). The reads pertaining to the enzyme phthalate 4,5-dioxygenase were only highlighted for the NA W samples (Figure 6), as well as for the enzyme 3-hydroxybenzoate 4-monooxygenase (Table S2 in Supporting Information); however, the latter was poorly recorded, and its result was not depicted in the barplot (Figure 6).

## 3. Discussion

The phyllosphere microbiome is increasingly recognized as an influential component of host plant biology from a metaorganism perspective and an extension of the ecosystem services offered by trees [17,51]. However, the influence of environmental factors on the stability of specific microorganism associations in the phyllosphere remains widely unclear, despite the extent of the phyllosphere habitat and the importance of this environment for plant ecology and biology [52,53]. This can be of particular interest for those species that are common in nature and widely used in urban forests, such as *Q. ilex*. Here, we provide the first characterization of both epiphytic fungal and bacterial communities colonizing the phyllosphere of *Q. ilex* across different environments located in the Campania region, Southern Italy.

Within this study, we first visualized microorganisms on the surface of leaves to highlight bacterial, as well as fungal, colonization hotspots. Such colonization hotspots were found in the proximity of micro-localized inhomogeneities coinciding with features of functional leaf traits, i.e., stomata, trichomes, and incised furrows of the cuticle (Figure 1), where higher nutrient availability due to physical accumulation may result in more favorable environments for colonization and proliferation [54]. In addition to the colonization of the adaxial leaf surface, the high levels of bacterial colonization at the stomata and trichomes at the abaxial surface are of great importance (Figure 1d–f). These structures represent an interface between the phyllosphere and the plant’s internal environment, where bidirectional flows of nutrients and chemical signals travel between the inside of the plant and the phyllospheric microenvironment and *vice versa*, establishing communication between the host and microorganisms [4]. The stomata, due to the mechanisms controlling their opening and closing, represent one of the first lines of physical defense to hinder pathogens from entering the leaves [55].

The environmental context in which the sampling and experimentation inherent to the definition of the phyllosphere of *Q. ilex* was performed is situated within the Mediterranean area [37]. This area is characterized by a peculiar alternation of aridity with a concomitant increase of temperatures in the summer, and an increase of precipitation in the autumn when temperatures decrease. These climatic features can influence the life cycle of plants and their associated organisms [56,57].

Emission sources of particulate matter are often more concentrated in urban areas (due to vehicular traffic and anthropogenic sources in general), leading to a greater exposure of plants to pollutants and a higher sequestration by leaf structures [58,59]. Our results were in line with this (Table 1). In addition, the variations of PM concentrations and retention on evergreen species’ leaves according to seasonality were higher in the winter than in the summer, in agreement with other authors [58,60].

Our data showed a shift in the microbial community mainly driven by seasonal factors (Figure 4A), as also found for other tree species (e.g., *Olea europea* L.) growing in a Mediterranean context [61]. Specifically, we observed that the winter season was characterized by a higher abundance of bacterial taxa in the phyllosphere, regardless of area type—urban or natural—and the resulting levels of air pollution (Figure 2 and Figure 3). Moreover, in the summer, we observed a drastic change of the phyllosphere colonization, which was dominated by fungal taxa (Figure 2 and Figure 3). The *Q. ilex* phyllosphere in the Mediterranean summer consists mainly (more than 60% of the total microbial community) of Ascomycota (Figure 2). The Mediterranean summer season characterized by long-term drought could have reduced the productivity of *Q. ilex* in some microbial populations, favoring fungal ones that are more resistant to external perturbations [62,63]. Our results are in line with previous results, which highlighted differing seasonal patterns among phyllospheric fungal taxa [64,65]. In fact, changes in the climate (water, temperature, light) imposed by seasonality were also associated with changes in plant productivity and, thus, in the supply of resources (water, nutrients, and organic matter). In particular, Ascomycota is the most diverse fungal group, living by various nutritional modes and showing remarkable tolerance to external perturbation [13]. Our data indicated an increase in microbial diversity, on a seasonal basis, linked to the higher presence of fungal taxa in the summer (Figure 2 and Figure 3), which, considering the Mediterranean context, could be explained by fungal life cycles or environmental tolerances [66]. Stone and Jackson [67] investigated a different species of the *Quercus* genus, outlining a great diversity and divergence between urban and non-urban stands, which is not fully consistent with our observations. This difference could be due to the peculiarities of the Mediterranean climate. Nevertheless, other authors [17] have also hypothesized and demonstrated the existence of a dominant phyllosphere microbiome that is persistent among geographically distant populations. Across samples and with the most stringent criterion (co-occurrence), we identified a core microbiome in the collective *Q. ilex* phyllosphere. We found that more than 78% of the microbial taxa co-occur among samples, suggesting a high stability. This finding is relevant, given the environmental heterogeneity of the sampling sites, and supports our hypothesis that the *Q. ilex* phyllosphere microbiome exhibits specific host association, as recognized for other plant species [17].

We also observed cross-domain (fungal–bacterial) connections (Figure 5), as also found by previous studies [68], suggesting the importance of interdomain phylogenetic diversity in microbiome assembly. Within this study, we also identified keystone taxa that include Lecanoromycetes (an Ascomycota class), which is an epiphytic foliar fungal taxon (Figure 5). This finding highly underpins the hypothesis that the presence of lichenized fungi can be a major shaper of the leaf microbiome, as rarely reported. The microbial network analyses also depict possible associations involving the interplay between certain classes of bacteria, such as phosphorus- and nitrogen-mobilizing bacteria (in our case-study, represented by Actinobacteria; Figure 5) and lichenized fungi, which is of fundamental importance in the leaf microbiome [2,69]. Kiritimatiellaeota, which is a recently proposed bacterial phylum [70] occurring in extreme environments [71] and also found in microbial communities of seagrass leaves [72], also represents a keystone species of the *Q. ilex* phyllosphere (Figure 5). In addition, based on our analyses, we supposed a key role of Firmicutes for the *Q. ilex* phyllosphere (Figure 5), which can be crucial as they are also well adapted to higher temperatures due to their ability to form spores [73,74].

Gomes et al. [59] investigated the specific composition of the epiphytic and endophytic fungi of leaves of *Olea europaea*, a species native to the Mediterranean area and, like *Q. ilex,* with adaptive leaf characteristics related to sclerophyll, such as a dense trichome layer, ticker cuticle, and waxy leaf surface. Comparing the epiphytic fungal taxa found in *O. europaea* during the spring-summer period [61] with those found in the same season on *Q. ilex* reveals an impressive overlap of the most present taxa (e.g., Dothideomycetes, Agaricomycetes, Sordariomycetes, and Eurotiomycetes) [61]. This convergence could suggest a potential phenomenon of ancient co-evolution (which needs to be further investigated) between sclerophyllous plant species, adapted to the Mediterranean climate [37], and specific groups of fungal (and bacterial) taxa colonizing leaf surfaces characterized by peculiar functional traits, reinforcing the concept of the holobiont [13,45,75].

Focusing on the potential microbial functions related to PAHs degradation, we explored functional metagenomics and considered the abundance of genes coding for key enzymes of PAH catabolic pathways [16]. Our data revealed that only the bacterial communities on the *Q. ilex* leaves harbored genes involved in the degradation of such pollutants (Table S2 in Supporting Information). The taxonomic identification of these PAHs-degrading genes is represented by some taxa of the core microbiome, such as Actinobacteria, which are mainly related to protocatechuate 3,4-dioxygenase. The latter catalyzes the aromatic ring cleavage of 3,4-dihydroxybenzoate and is involved in PAHs degradation pathways [76]. Actinobacteria are well known to have a variety of degradation substrates, especially xenobiotic organic compounds [77]. Several genes found in the *Q ilex* phyllosphere belong to Alpha-, Beta-, and Gamma-Proteobacteria, which are also involved in the degradation of PAHs [78].

We assume that the observed data concerning the abundance of PAHs-degraders, which is higher in the winter season (natural area), may be mainly related to not only pollutant concentrations, but also seasonal variations of other parameters (as previously suggested by Franzetti et al. [16]) that were not explored in this work. Upcoming research should be conducted to identify possible correlations between foliar morphology and chemistry, as well as the dynamics of phyllosphere communities over environmental factors (e.g., seasons), while also searching for co-evolutionary patterns between the adaptation strategies to climatic conditions of higher plants and the microorganisms constituting their phyllosphere.

## 4. Conclusions

In this study, we explored the phyllosphere microbiome, which is relevant as an extended plant phenotype termed the plant holobiont [75]. We reported for the first time, the intra- and inter-domain phyllosphere features of *Q. ilex*. in a Mediterranean context, highlighting its composition and variation under seasonal influence. We concluded that the microbial diversity of the phyllosphere is particularly sensitive to the changes of climatic factors occurring throughout the seasons and that urban and non-urban patterns (considered as concentrations of airborne pollutants) may exert a co-effect. We also found that the phyllosphere of *Q. ilex* harbors microbial potential for PAH degradation, as demonstrated by 83 identified genes coding for enzymes involved in PAH degradation pathways. The provided new data can be a useful knowledge base to improve our understanding of *Q. ilex* phyllosphere community dynamics, which could be valuable to predict their responses to a changing climate, and relevant for the host, as they can strongly influence its behavior.

These research directions can also be a blueprint in the context of next-generation biomonitoring, for detecting and explaining shifts in the relationships between plants and microorganisms under environmental pressure.

## 5. Materials and Methods

### 5.1. Sites Description and Sample Collection

A preliminary identification of sampling sites in urban and natural areas was made. The urban areas (UA) considered fell within the city boundaries of Naples (Southern Italy, 40.8518° N, 14.2681° E), within which rows or groups of *Q. ilex* trees, located near roads with high vehicular traffic, were identified. The natural area (NA) sampling locations were located in *Q. ilex* woodland within the administrative boundaries of Ottati municipality (Southern Italy, 40.4646° N, 15.3085° E), which is within the Cilento, Vallo di Diano e Alburni National Park [79]. The soil types are Eutric Vertisols (calcaric) e Calcic Vertisols and Epileptic Andosols (Eutric) (based on the World Reference Base for Soil Resources-WRB classification) for UA and NA, respectively. In 2020, the mean temperatures were 11.1 °C and 9 °C (in winter season) and 23.8 °C and 20.4 °C (summer season) for UA and NA, respectively. As concerns annual precipitation, in 2020, 116.8 mm and 116.9 mm were recorded in UA and NA, respectively. The NA sampling locations were at least 1 km away from vehicular roads. Both urban and natural sampling sites were chosen based on proximity to the Campania Regional Environmental Protection Agency (ARPAC) air monitoring stations [80]. For the urban and natural areas, three and two sampling sites, respectively, were identified following the above-mentioned criteria. Three trees spaced a maximum of 4 m apart were selected from each area (9 trees from UA and 6 from NA in total). For quantification of airborne pollutants on the leaf surfaces, sampling procedures proposed by Sgrigna et al. (2015) [35] were used: leaves were collected from branches at two different heights, from the highest and the lowest part of the canopy. Leaves from each branch were cut with metal scissors and placed in plastic bags, representing a leaf area of about 1500 cm^2^ in total. Leaves from each sampling site were mixed to obtain homogenous samples representative of each UA and NA site. For phyllosphere metagenomic analysis, sampling procedures described by Franzetti et al. [16] were followed, with a few modifications (e.g., number of leaves). Eight leaves per tree were collected at an average height ranging approximately between 1.5 and 2 m. Leaves were cut from branches using metal scissors and tweezers, both sterilized each time with ethanol, and immediately put in sterile plastic bags to prevent any exogenous DNA contamination. Both samplings were carried out at two different times of the year: in winter (W, in February) and in summer (S, in July). DNA extractions were performed within 48 h from sampling plant material, preserving the cold chain of samples at 4 °C. After the DNA extraction, leaves were air-dried, and leaf surfaces, from the samples collected in each season and at each sampling station, were measured through scanning and subsequent area calculation using ImageJ software.

### 5.2. Two-Photon Excitation Microscopy (TPEM) Analyses

To visualize epiphytic microorganism aggregations on *Q. ilex* leaves and how they correlated topologically with the leaf surface functional traits of the host species (cuticle, trichomes, and stomata), two-photon excitation microscopy observations were performed using Syto 9 and propidium iodide (LIVE/DEAD BacLight Bacterial Viability Kit-Invitrogen) as staining fluorescent dyes [4]. Staining was performed to highlight nucleic acids in the cells of the microorganisms, allowing the localization of the bacteria and fungi colonization on the *Q. ilex* leaves at their adaxial and abaxial surfaces. The dense coverage of stellate trichomes on the abaxial side impeded good visualization of the phyllosphere microorganisms; hence, the dense trichome layer was partially removed using adhesive tape, according to the methodology described in Prigioniero et al. [37]. The two dyes were used to visualize the living cells of the phyllospheric microorganisms in green fluorescence and the dead cells of the microorganisms in red fluorescence. Steps for phyllosphere microorganism staining included the mixing of equal volumes of Syto9 and propidium iodide in a microfuge tube. A total of 10 µL of dye mixture volume was added to a 0.5 cm^2^ of leaf surface and incubated at room temperature in the dark for 15 min, then mounted using mounting oil and coverslip. Microorganisms were visualized based on spectral properties of both dyes: 480/500 nm and 490/635 nm (excitation/emission) for Syto9 and propidium iodide, respectively. Images were observed and acquired using a two-photon excitation microscope (Ultima investigator 2-photon microscope–Bruker, MS, Billerica, MA, USA).

### 5.3. PM_10_ Extraction and Quantification on Leaf Surfaces

Extraction of coarse particulate matter (PM_10_) was performed with the methodology proposed by Dzierzanowski et al. [33]. About 400 cm^2^ of leaves was used. After an initial washing of the leaves with distilled water to remove particulate matter deposited on the surface, the resulting suspension was vacuum filtered using pre-weighted, stabilized filters (filter paper grade 91, Whatman™) to retain PM_10_. After filtration, the filters were placed in a drying oven at 60 °C for 30 min, weighed, and the difference in weight was recorded. The operation was repeated with the same leaves, using chloroform to extract the epicuticular waxes and particulate matter present in them [33,37]. Data were expressed as ng cm^−2^ of PM_10_ related to leaves surfaces washed. The total amount of PM_10_ retained was calculated as the sum of the weighted filters obtained from the water and chloroform washings. Each analysis was performed in triplicate.

### 5.4. PAHs Associated to PM_10_ Chemical Analyses

A mixture of acetone-hexane (60:40 *v/v*) was added to each filter containing the particulates from the previous filtrations and extracted using an ultrasonic extractor for 10 min (Sonica, SOLTEC, Milan, Italy). The extract was concentrated to a final volume of 1 mL by evaporation under nitrogen flow (MultiVap 10, Labtech, Bergamo, Italy), cleaned on a sulphate sodium anhydrous column. Then, 10 µL of the internal standard was added (mixture of 5 deuterated PAHs at the concentration of 10 mg L^−1^) and injected into a gas chromatograph (GCMS-TQ8030 Shimadzu, Kyoto, Japan) coupled with a triple quadrupole mass spectrometer (MS-TQ8030, Shimadzu, Kyoto, Japan) and a fused silica HP5-MS capillary column (30 m length × 0.25 mm inner diameter, film thickness 0.25 μm; Agilent Technologies, US). The separation was conducted with the oven temperature programmed as follows: initial setting at 80 °C (2 min hold), ramped to 180 °C at 20 °C min^−1^, and finally to 300 °C at 5 °C min^−1^ (9 min hold). The injector was held at 280 °C. The mass spectrometer was operated in SIM (selected ion monitoring) mode. The identification of PAHs in the solutions extracted was done based on previously determined retention times, and it was confirmed using mass spectra of the NIST library (NIST 20, 2020). The quantification of PAHs was performed using an external calibration curve together with the internal standards. The detection limit (LOD) and limit of quantification (LOQ) for each investigated PAH were calculated using the range method of prediction to 95% of linear regressions. The calculated averages of LOD and LOQ were 0.007 μg m^−2^ and 0.02 μg m^−2^ (surface referred to the area of the leaves from which PM was extracted). The data quality is ensured by certified reference materials (CRM-NIST 3253), and the recovery percentage was estimated by analyzing in triplicate the CRM, and the observed range was 70–110%. The PAHs considered by the analysis included benzo[a]pyrene, benzo[a]anthracene, benzo[b]fluoranthene, benzo[j] fluoranthene, benzo[k]fluoranthene, indeno[1,2,3-cd]pyrene, and dibenzo[a,h]anthracene. Those are the carcinogenic compounds widely present in the urban airborne particulate matter [81]. Each analysis was performed in triplicate.

### 5.5. DNA Extraction for Taxonomic Analysis and Functional Profiling

The leaves placed in sealable sterile bags were washed with a 0.9 M NaCl solution, shaken, and placed in an ultrasonic bath for 15 min to release epiphytic microorganisms from the leaf surfaces. The washing water was then filtered on microbiological filters with a porosity of 0.45 um, as described by Gandolfi et al. (2017) [14]. Half of a filter was used for DNA extraction using the ZymoBiomics DNA Microbiome kit to obtain at least 1 ug of DNA per sample, required by the provider for the metagenomic analysis. The surfaces of the washed leaves were then measured with ImageJ (cm^2^).

### 5.6. Metagenomic Analyses of Bacterial and Fungal Communities

Metagenomics characterization of the summer and winter samples was performed by Whole Genome Sequencing. As a first step, a quality check was performed on the raw sequencing data, removing low-quality bases and adapters, while preserving the longest high-quality part of the reads. The minimum length established was 35 bp and the quality score 25, which increases the quality and reliability of the analysis. The quality of the reads was checked before and after the trimming step. The second step of the analysis was the taxonomic profiling and quantification of the samples. For this purpose, the software GAIA (v2.02-Sequentia Biotech, Barcelona, Spain) (https://metagenomics.sequentiabiotech.com/gaia/, accessed on 1 October 2022) was used. The software works as follows: (1) each pair of reads is aligned against one or more reference databases, and the best alignments are extracted; (2) a Lowest Common Ancestor (LCA) algorithm is applied to the best alignments; (3) identity and coverage thresholds are applied to the alignments; (4) taxonomy is summarized and reported. Identification of pathways involved in the degradation of hydrocarbons was performed by first aligning the reads against UniRef90 (release 2021_03) using diamond (https://github.com/bbuchfink/diamond, accessed on 1 October 2022) following the FAMLI pipeline (https://github.com/FredHutch/FAMLI, accessed on 1 October 2022). Enzymes involved in the KEGG pathway called “Polycyclic aromatic hydrocarbon degradation” [50] were isolated using the KEGG pathway code map00264 (https://www.genome.jp/kegg-bin/show_pathway?map00624, accessed on 1 October 2022), and the number of reads associated with each protein were obtained from the FAMLI results.

Differential Abundance Analysis (DAA) was performed using ZicoSeq (*GUniFrac* package in R environment) [82]. ZicoSeq is a linear model and permutation-based method for differential abundance analysis of zero-inflated compositional sequencing data (such as microbiome sequencing data). ZicoSeq was chosen because it: (i) accounts for compositional effects, (ii) is useful when outliers with extremely large counts occur, and (iii) includes an empirical Bayes approach useful to account for zeros. DAA was used to identify Season (S and W) differential abundant OTUs, while adjusting the area type (U and N), because it is a potential confounder (based on our hypothesis).

### 5.7. Network Analysis of Phyllosphere Microorganisms

Network analysis was performed using R with psych package [83] and Gephi software [84]. The correlation matrix was constructed by calculating all possible pairwise Spearman’s rank correlations between OTUs, with more than ten sequences (146 OTUs) in order to remove poorly represented OTUs. We considered a valid co-occurrence event to be a robust correlation if the correlation coefficient (ρ) was both >0.6 or <−0.6 and statistically significant (*p*-Value < 0.05; [85]). The Gephi software was used to visualize the bipartite network graphs using the Fruchterman–Reingold algorithm [86]. The nodes in the reconstructed network represent the OTUs, whereas the edges (which are the connections) correspond to a strong and significant correlation between nodes [87]. Further, we used modularity analysis to identify the microbial community structure and depict groups of nodes with dense connections within groups and sparser connections between groups. For each node, the betweenness centrality (BC) was calculated to identify keystone species within community networks. BC measures the influence of a node on all other relationships between nodes in the network, and high BC values indicate more important roles in the network [88]. A Venn diagram was generated to show the shared and unique OTUs among groups, based on the occurrence of OTUs in the samples, regardless of their relative abundance [89]. A heatmap was constructed to show the most abundant OTUs, with abundance >75th percentile relative to sample type.

## Figures and Tables

**Table 1:** Average concentrations (±SE) of coarse particulate matter (PM_10_) and six polycyclic aromatic hydrocarbons (PAHs) extracted with water and chloroform from the leaves of *Q. ilex* sampled in urban areas (UA) and natural areas (NA) during the winter and summer.

	PM_10_ (ng cm^−2^)		Benzo(a)pyrene (ng cm^−2^)		Benzo(a)anthracene (ng cm^−2^)		Benzo(b)fluoranthene (ng cm^−2^)		Benzo(j)fluoranthene (ng cm^−2^)		Benzo[k]fluoranthene (ng cm^−2^)		Indeno[1,2,3-cd]pyrene (ng cm^−2^)		Dibenz[a,h]anthracene (ng cm^−2^)	
	H_2_O	ClCH_3_	H_2_O	ClCH_3_	H_2_O	ClCH_3_	H_2_O	ClCH_3_	H_2_O	ClCH_3_	H_2_O	ClCH_3_	H_2_O	ClCH_3_	H_2_O	ClCH_3_
UA Winter	91,628.54 ± 959.47 ^a^	38,926.46 ± 755.81 ^abc^	0.47 ± 0.12	0.1 ± 0.05 ^a^	nd	nd ^a^	0.09 ± 0.04 ^a^	nd ^a^	0.09 ± 0.05	nd	nd	nd	nd	nd ^a^	nd	nd
NA Winter	37,789.85 ± 4549.16 ^b^	21,022.88 ± 1443.39 ^a^	0.42 ± 0.19	0.36 ± 0.16 ^ab^	nd	0.12 ± 0.05 ^b^	nd ^b^	nd ^a^	nd	nd	nd	nd	nd	nd ^a^	nd	nd
UA Summer	91,375.23 ± 4978.18 ^a^	26,908.81 ± 1274.22 ^b^	0.49 ± 0.05	0.1 ± 0.05 ^a^	nd	nd ^a^	nd ^b^	nd ^a^	nd	nd	nd	nd	nd	nd ^a^	nd	nd
NA Summer	75571.41 ± 2889.44 ^ab^	27,465.91 ± 6274.23 ^c^	0.55 ± 0.25	0.72 ± 0.05 ^b^	nd	0.15 ± 0.07 ^a^	0.19 ± 0.08 ^b^	0.17 ± 0.08 ^a^	nd	nd	nd	nd	nd	0.28 ± 0.13 ^a^	nd	nd

Symbols as: nd—not detected. Different letters indicate significant differences among samples (UA *n* = 9; NA *n* = 6; *p* < 0.05).

## Data Availability

Data is contained within the article or supplementary material.

## Supplementary Materials

The following supporting information can be downloaded at: https://www.mdpi.com/article/10.3390/plants11243528/s1, Figure S1: Modularity classes of phyllosphere microbiome of *Q. ilex*; *x*-axis shows the number of modularity class, *y*-axis show the number of OTUs falling into each modularity class; Table S1: Correlations and topological properties of microbiome networks from phyllosphere of *Q. ilex*.; Table S2: Functional metagenomic data (Kegg pathway code: ec00624); number of reads per samples are shown.
