# Supplementary material for: Quercus ilex Phyllosphere Microbiome Environmental-Driven Structure and Composition Shifts in a Mediterranean Contex
Source: Plants (Basel). 2022 Dec 15;11(24):3528. doi: 10.3390/plants11243528 (PMC9782775; doi:10.3390/plants11243528)
Supplement: Supplementary file 1 [file plants-11-03528-s001.zip › Supporting Information.pdf]

Supporting Information

Quercus ilex phyllosphere microbiome environmental-driven structure and composition shifts in a Mediterranean contex

Alessia Postiglione[1a], Antonello Prigioniero[1a], Daniela Zuzolo[1*], Maria Tartaglia[1], Pierpaolo Scarano[1], Maria Maisto[1], Maria Antonietta Ranauda[1], Rosaria Sciarrillo[1], Sofie Thijs[2], Jaco Vangronsveld[2,3], Carmine Guarino[1]

[1] Department of Science and Technology, University of Sannio, via de Sanctis snc, Benevento 82100, Italy

[2] Environmental Biology, Centre for Environmental Sciences, Hasselt University, Agoralaan, building D, 3590 Diepenbeek, Belgium

[3]Department of Plant Physiology and Biophysics, Institute of Biological Sciences, Maria Curie-Skłodowska University, Akademicka 19, 20-033 Lublin, Poland;

[a] equal contribution

* author for correspondence dzuzolo@unisannio.it

Fig. S1 Modularity classes of phyllosphere microbiome of Q. ilex; x-axis shows the number of modularity class, y-axis show the number of OTUs falling into each modularity class

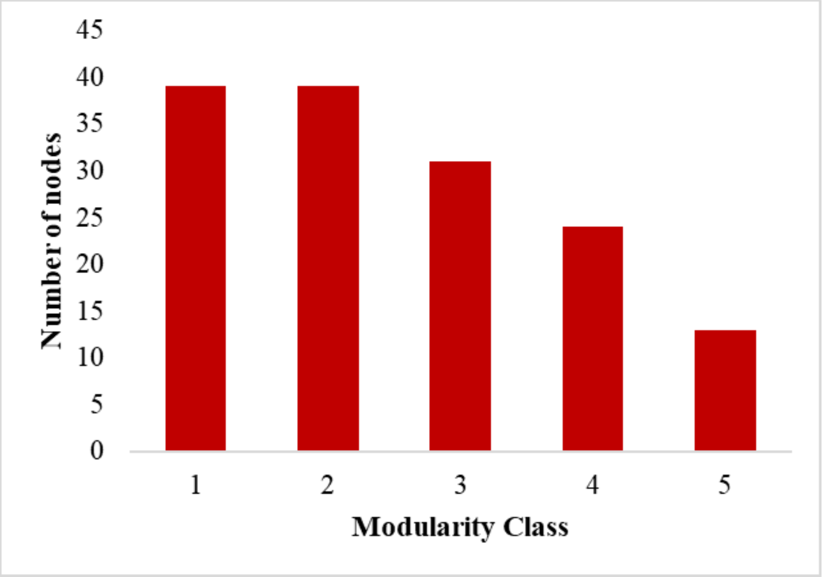

Table S1 Correlations and topological properties of microbiome networks from phyllosphere of Q. ilex.

Network properties	
Number of nodes[a]	146
Number of edges[b]	3,164
Modularity[c]	0.44
Number of communities[d]	4
Network diameter[e]	5
Average path length[f]	2.34
Average degree[g]	21.62
Av. clustering coefficient[h]	0.614
Positive edges[i]	2,532
Negative edges[l]	632

[a]Microbial taxon (at ASV level) with at least one significant ($P < 0.05$) and strong (Spearman's correlation coefficient > 0.8 or < −0.8) correlation;
[b]Number of connections/correlations obtained by Spearman analysis;
[c]The capability of the nodes to form highly connected communities, that is, a structure with high density of between nodes connections (inferred by Gephi);
[d]A community is defined as a group of nodes densely connected internally (Gephi);
[e]The longest distance between nodes in the network, measured in number of edges (Gephi);
[f]Average network distance between all pair of nodes or the average length off all edges in the network (Gephi);
[g]The average number of connections per node in the network, that is, the node connectivity (Gephi);
[h]How nodes are embedded in their neighborhood and the degree to which they tend to cluster together (Gephi).
[i]Spearman positive correlation (> 0.5 with $P < 0.05$);
[l]Spearman negative correlation (< −0.5 with $P < 0.05$).

Table S2 Functional metagenomic data (Kegg pathway code: ec00624); number of reads per samples are shown [Table S2 is an excel file submitted separately]
